# Supplementary material for: The interactome of intact mitochondria by cross-linking mass spectrometry provides evidence for coexisting respiratory supercomplexes
Source: Mol Cell Proteomics. 2017 Dec 8;17(2):216–32. doi: 10.1074/mcp.RA117.000470 (PMC5795388; doi:10.1074/mcp.RA117.000470)
Supplement: Supplemental Data [file supp_RA117.000470_133922_0_supp_23978_fzffwf.zip › spectra_annotation/mito_DR_spectra_annotation/49-1-4-1-1-1.pdf]

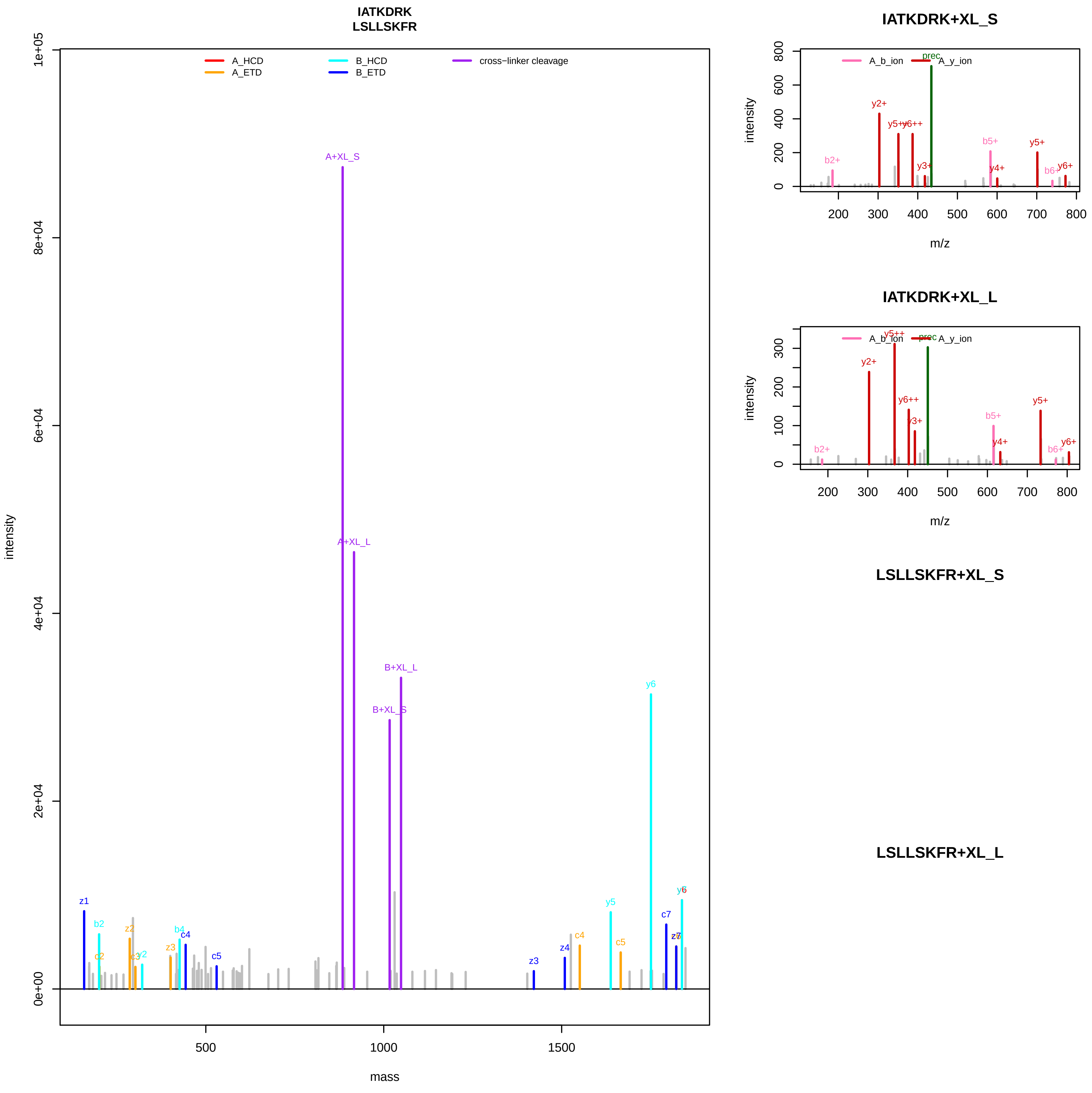

IATKDRK
LSLLSKFR
A_HCD
A_ETD
B_HCD
B_ETD
cross−linker cleavage
A+XL_S
A+XL_L
B+XL_L
B+XL_S
y6
y5
z1
b2
c2
z2
c3
y2
z3
b4
c4
c5
z3
z4
c4
c5
c7
z7
intensity
mass
0e+00
2e+04
4e+04
6e+04
8e+04
1e+05
500
1000
1500
IATKDRK+XL_S
A_b_ion
A_y_ion
prec
y2+
y5++
y6++
b2+
y3+
b5+
y4+
y5+
b6+
y6+
intensity
m/z
IATKDRK+XL_L
LSLLSKFR+XL_S
LSLLSKFR+XL_L
